# Effect of Annealing on the Surface Hardness of High-Fluence Nitrogen Ion-Implanted Titanium

**DOI:** 10.3390/ma16103837

**Published:** 2023-05-19

**Authors:** Petr Vlcak, Josef Sepitka, Jan Koller, Jan Drahokoupil, Zdenek Tolde, Simon Svoboda

**Affiliations:** Faculty of Mechanical Engineering, Czech Technical University in Prague, Technicka 4, 16607 Prague, Czech Republic; josef.sepitka@fs.cvut.cz (J.S.); jan.koller@fs.cvut.cz (J.K.); jan.drahokoupil@fjfi.cvut.cz (J.D.); zdenek.tolde@fs.cvut.cz (Z.T.); simon.svoboda@fs.cvut.cz (S.S.)

**Keywords:** titanium, nitrogen ion implantation, hardness

## Abstract

Commercially pure titanium grade II was kinetically nitrided by implanting nitrogen ions with a fluence in the range of (1–9)·10^17^ cm^−2^ and ion energy of 90 keV. Post-implantation annealing in the temperature stability range of TiN (up to 600 °C) shows hardness degradation for titanium implanted with high fluences above 6·10^17^ cm^−2^, leading to nitrogen oversaturation. Temperature-induced redistribution of interstitially located nitrogen in the oversaturated lattice has been found to be the predominant hardness degradation mechanism. The impact of the annealing temperature on a change in surface hardness related to the applied fluence of implanted nitrogen has been demonstrated.

## 1. Introduction

Titanium and titanium alloys are frequently used in aircraft, chemical and biomedical applications due to their high yield strength, high corrosion resistance, low density and low elastic modulus [1,2]. However, undesirable friction, wear and surface hardness are the main disadvantage of titanium materials, especially when there is contact between mutually moving mechanical parts [3,4,5]. Nitridation is an effective approach for forming a continuous hard TiN barrier layer on the surface integrated into the Ti matrix without the risk of delamination. The high process temperatures of conventional and innovative nitriding techniques tend to be limiting for materials with a temperature-sensitive structure [6]. However, nitrogen ion implantation, an advanced kinetic form of nitriding, is a promising technique for low-temperature processing with controllable depth distribution of nitrogen. Schmidt [7] and other researchers [8,9] have shown the beneficial effect of implanting nitrogen with an accelerating voltage up to 250 kV and fluences up to 2·10^18^ cm^−2^ on the surface characteristics of α- and α + β-Ti alloys. Significantly improved surface hardness, friction resistance and corrosion resistance at fluences above 10^17^ cm^−2^ have been observed. The improved surface properties are brought about by finely dispersed TiN with a crystallite size between 3 and 50 nm [10,11]. TiN is formed along the concentration depth profile in the region where the N concentration exceeds the solubility limit in Ti [10,12]. Below the solubility limit, nitrogen located in the Ti lattice gives rise to an α-Ti(N) solid solution, which is a dominant hardening mechanism at lower fluences [7]. Research in this field has shown a beneficial impact of elevated temperature during ion implantation on the thickness of the modified area, as well as additional formation of TiN in the regions with a diffusion-enhanced concentration of nitrogen. Manova [13], Oliveira [14] and Silva [15] found that ion implantation at elevated temperatures up to 900 °C resulted in a thicker modified zone in which the hardness, friction coefficient, wear resistance and corrosion resistance of pure titanium and of Ti-6Al-4V alloy were improved. In contrast, post-implantation annealing can also have an effect on the microstructure of the modified layer and on its surface hardness with regard to the temperature stability of the predominant hardening mechanism [16]. Recent research has suggested that annealing in the temperature stability range of TiN can lead to hardness degradation [17]. The temperature sensitivity of the interstitial solid solution Ti + N and its redistribution in the structure was considered to be the hardness degradation mechanism. The interstitial solid solution is preferentially formed in nitrogen-deficient regions. Therefore, the impact of post-implantation annealing may vary depending on the fluence of the nitrogen ions, which determine the resulting concentration profile and limit the concentration of nitrogen for phase formation. Many articles have been published on nitrogen implantation at elevated temperature, but to the best of our knowledge, there has been no study on the effect of post-implantation annealing on the microstructure and on surface hardness as a function of nitrogen ion fluence. This paper reports on the effect of post-implantation annealing on the hardness stability of nitrogen ion-implanted titanium. The evolution of surface hardness with respect to ion fluence and annealing temperature is investigated.

## 2. Materials and Methods

In this study, samples 5 mm in height and 14 mm in diameter were made of commercially pure titanium grade 2 (Tirus International SA., Lausanne, Switzerland). Before nitridation, the surface of the samples was ground with abrasive plates in the range from 80 to 2500 grit. Finishing to a mirror-like surface was performed with 1 µm diamond paste.

Nitrogen ions were implanted at an accelerating voltage of 90 kV with fluences of 1, 3, 6 and 9∙10^17^ cm^−2^. Applied fluences express the proportional increase in the number of implanted ions with the fluence number. An ion beam incidence angle of 90° was used. A process temperature below 100 °C was maintained by controlling an ion current which was below 2 µAcm^−2^. After nitrogen implantation, the samples were annealed at temperatures of 400 °C and 600 °C. The post-implantation annealing was carried out in a vacuum chamber at a pressure of 2∙10^−3^ Pa. The annealing time was equal to the implantation time: 1 h for fluence 1∙10^17^ cm^−2^, 3 h for fluence 3∙10^17^ cm^−2^, 6 h for fluence 6∙10^17^ cm^−2^ and 11 h for fluence 9∙10^17^ cm^−2^. The same annealing time was used for both 400 and 600 °C.

The nitrogen concentration profiles were measured by Glow Discharge Optical Emission Spectroscopy (GD-OES). The phase composition was investigated by X-ray diffraction (XRD), and morphological characterization was performed by a scanning electron microscope (SEM). The surface hardness was measured by the nanoindentation technique. A continuous measurement mode (CMX) was applied to obtain the hardness depth profiles. The CMX indentation function was prescribed by a quasistatic force (Pqstat) in the range from 0.5 to 10,000 μN. The dynamic actuation force (Pdyn) was prescribed in the range from 1 to 144.7 μN at a frequency of 200 Hz. For each sample, average values were obtained from 16 indents in a 4 × 4 matrix with a separation step of 5 μm. The measurements were performed at a temperature of 23 °C.

## 3. Results and Discussion

**Figure 1:**
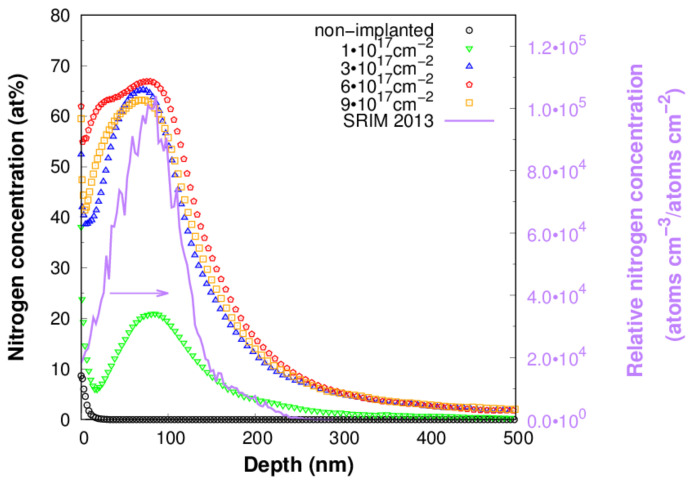
Depth concentration profiles of nitrogen measured by GD-OES on titanium implanted with fluences of 1, 3, 6 and 9·10^17^ cm^−2^ (open symbols) and depth distribution calculated by the SRIM 2013 code (full purple line). SRIM-computed depth distribution of nitrogen is plotted in relative concentration (left purple *y*-axis) for clear and easy comparison with experimental concentration profiles measured on samples implanted with various fluences.

**Figure 2:**
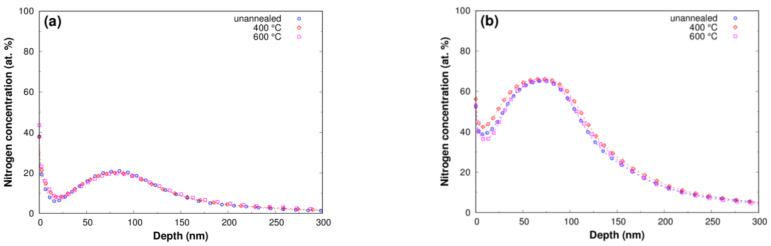

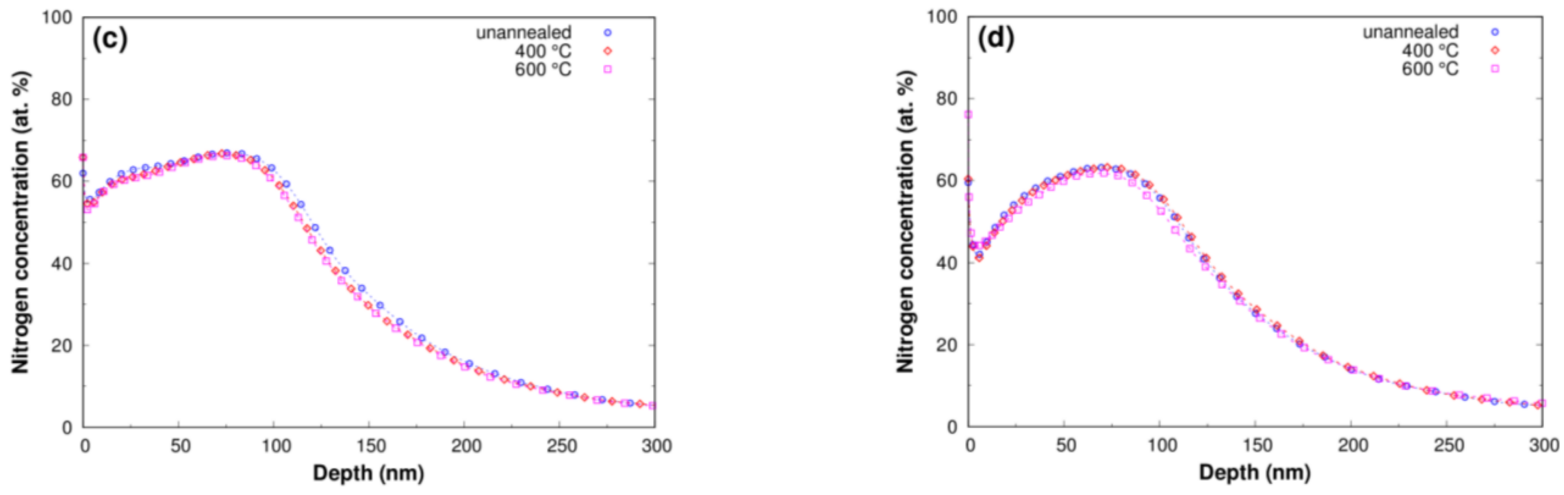
Depth concentration profiles of nitrogen measured by the GD-OES method on titanium samples implanted with fluences of (**a**) 1∙10^17^ cm^−2^, (**b**) 3∙10^17^ cm^−2^, (**c**) 6∙10^17^ cm^−2^ and (**d**) 9∙10^17^ cm^−2^ and post-implantation annealed at temperatures of 400 °C and 600 °C.

Figure 1 shows typical nitrogen concentration profiles measured by the GD-OES method on Ti samples implanted at various fluences. The maximum N concentration of approx. 21 at% was found at a depth of approx. 76 nm for a fluence of 1∙10^17^ cm^−2^, the maximum concentration of approx. 65 at% was at a depth of approx. 75 nm for a fluence of 3∙10^17^ cm^−2^; the maximum concentration of approx. 67 at% was at a depth of approx. 79 nm for a fluence of 6∙10^17^ cm^−2^; and the maximum concentration of approx. 63 at% was at a depth of approx. 73 nm for a fluence of 9∙10^17^ cm^−2^. The measured depth distributions are in good agreement with the simulated N depth distribution from the SRIM2013 code [18] (full purple line in Figure 1). A comparison of the N concentration profiles measured on annealed and unannealed Ti samples indicates a minor effect of annealing on the depth distribution of N (Figure 2). No detectable change in concentration profiles indicating diffusion of nitrogen after annealing was observed. The sensitivity of the measurement method that is used can undoubtedly have an effect on this. Hydrogen corrections were used to process the measured signal intensity, enabling precise depth profiling at a shallow depth near the surface [19]. GD-OES shallow depth profiling has been successfully used by other researchers for nitrogen ion-implanted titanium [20]. Annealing times and annealing temperatures may be other reasons why nitrogen diffusion did not occur. Although the annealing times correspond to the time of implantation, deformation of the concentration profile did not appear even at the highest fluence, which took place for a period of 11 h. The maximum concentration of nitrogen for applied fluences of 3, 6 and 9∙10^17^ cm^−2^ is above 50 at%. This nitrogen concentration is higher than the equilibrium concentration of nitrogen in TiN and leads to α-titanium lattice oversaturation. The results in Figure 1 indicate that a fluence higher than 6∙10^17^ cm^−2^ leads to a decrease in maximum concentration, which is associated with nitrogen effusion. Nitrogen effusion and a decrease in maximum nitrogen concentration were also observed in a TiNbZrTa β-Ti alloy ion implanted with fluences leading to oversaturation [21].

**Figure 3:**
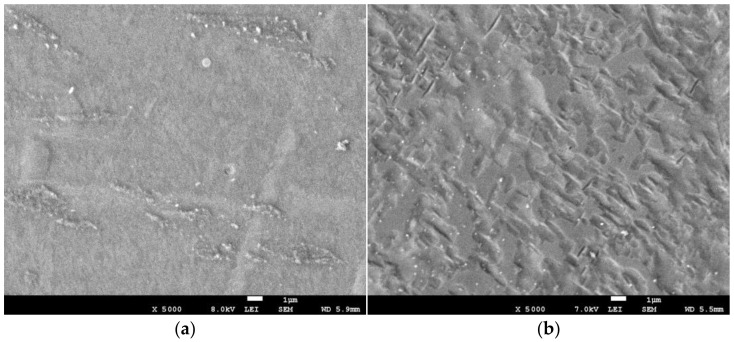

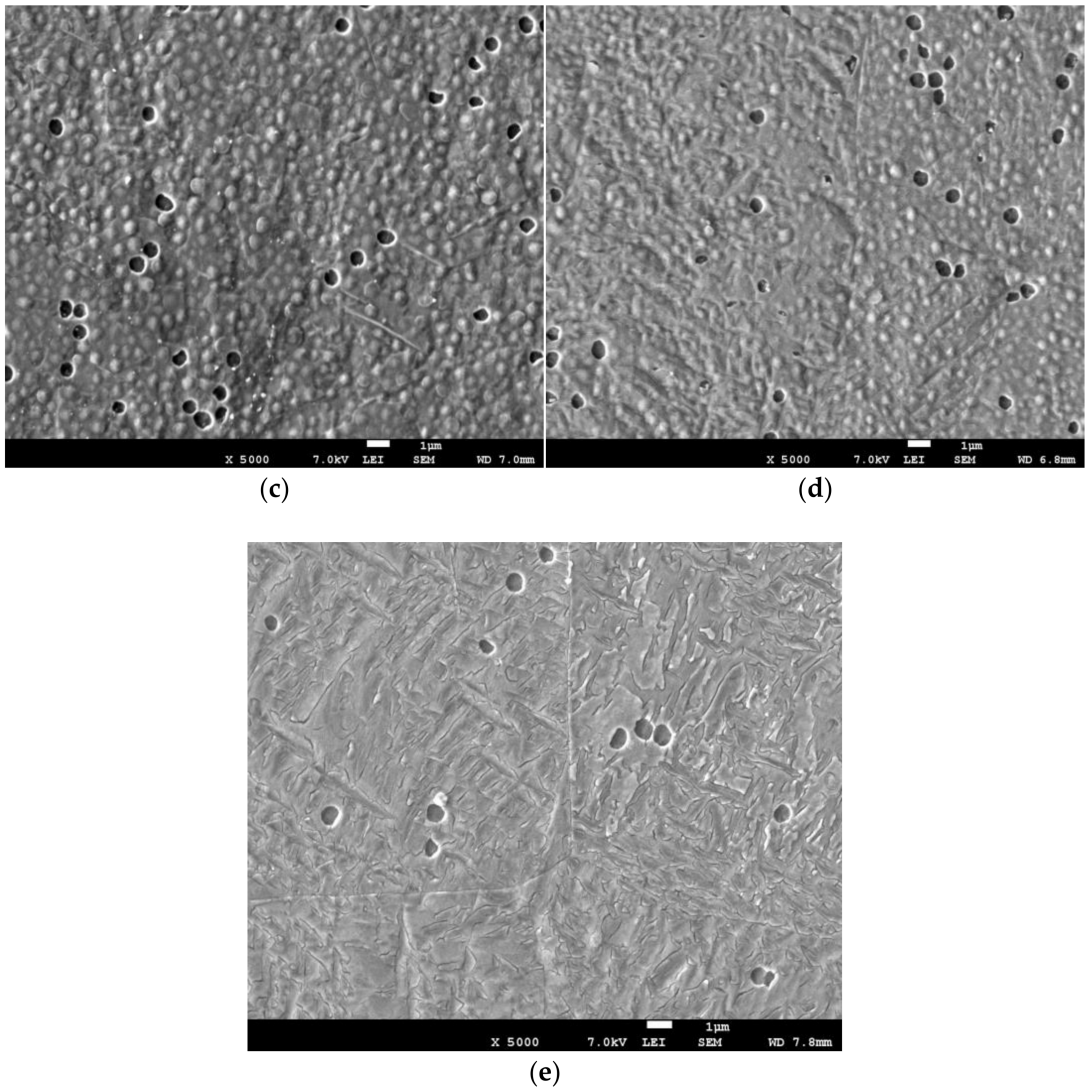
SEM top view micrographs of non-implanted titanium (**a**) and titanium implanted with nitrogen ions with fluences of (**b**) 1∙10^17^ cm^−2^, (**c**) 3∙10^17^ cm^−2^, (**d**) 6∙10^17^ cm^−2^ and (**e**) 9·10^17^ cm^−2^.

There are observable signs of oversaturation (saturation defects) on the surface morphology. The evolution of saturation defects (blisters, circular craters and cracks) with applied fluence can be seen on the SEM top view micrographs in Figure 3. Samples implanted with fluence 1∙10^17^ cm^−2^ showed no sign of saturation defects (Figure 3b). An only slightly etched surface morphology after ion sputtering during ion implantation can be seen, without other defects. Surface blistering appears on the titanium surface implanted at fluence 3 and 6∙10^17^ cm^−2^ (Figure 3c,d). Closed blisters are predominantly formed on the surface. The minor open circular craters indicate that some of the blisters are sputtered when these fluences are applied. Circular craters and cracks are well developed on the surface after ion implantation at the highest fluence of 9∙10^17^ cm^−2^ (Figure 3e). As expected, sputtering of the blisters led to the formation of craters without reforming the blisters. Massive titanium sputtering during N ion implantation was also observed by Li et al. [8] and by Aghajani et al. [10], but they reported no blistering. Surface blistering was observed on TiNbZrTa β-Ti alloy after nitrogen ion implantation at comparable ion energy and fluences [21]. A nitrogen ion implanted titanium surface with saturation defects may lead to lower corrosion resistance in a physiological saline solution. However, in the case of the TiNbZrTa alloy, it was found that the decreasing N concentration on top of the surface has a greater influence than surface saturation defects on reducing the corrosion resistance of the oversaturated Ti lattice [21].

The phase composition of an ion-implanted titanium surface was investigated by the XRD method under incident angles of 0.5° and 2°. These incident angles characterize the surface regions (material volume) given a depth of 149 nm for 0.5° and 571 nm for 2° [17] and were chosen in order to investigate the changes in phase composition of a shallow region and a deeper region of the modified surface. The results of a quantitative XRD phase analysis are presented in Table 1. Nitrogen-enriched surfaces are composed of the (hcp) α-Ti phase (ICSD #76265), the cubic (fcc) TiN (ICSD #1547) and Ti + N mixed interstitially located nitrogen in α-Ti (ICSD #108614). Ti+N was found to be the predominant structural phase for the sample implanted with fluence 1∙10^17^ cm^−2^. The concentration of Ti + N in the implanted titanium surface decreased with the increasing fluence. This phenomenon is caused by the increased surface concentration of nitrogen (Figure 1) above its solubility in α-Ti, which gives rise to TiN [12]. TiN is the predominant structural phase in both the shallow and the deeper surface regions (incident angles 0.5° and 2° in Table 1) for fluence 3∙10^17^ cm^−2^ and higher. It is demonstrated well that a higher concentration of TiN is detected in the shallow region than in the deeper region (Figure 1). Ti + N shows the opposite trend. These findings are consistent with the depth distribution of nitrogen in Figure 1 and Figure 2 and demonstrate the changing phase concentration of the structural composition with a variable nitrogen concentration along the concentration profile. The quantitative phase analysis assumes homogeneous distribution of the phases. In fact, however, the distribution of the phases is inhomogeneous. For this reason, the proportion of the phases that are located closer to the surface is overestimated. Depending on the distribution of the phases, the relative error of the evaluation method is 0.5–1.5%. X-ray radiation decreases exponentially in the material. The results in Table 1 therefore represent average values obtained from a layer of a given thickness.

The results of XRD phase analysis in Table 1 indicate that post-implantation annealing affects the phase composition of the modified surface for fluence 3, 6 and 9∙10^17^ cm^−2^, where signs of oversaturation were observed. In the deeper region (incident angle 2°), a decreasing tendency in Ti + N concentration with the temperature is observed. Conversely, in the region closer to the surface (incident angle 0.5°), the concentration of Ti + N shows an increasing tendency. The concentration of TiN is rather stable, with increasing temperature in both measured regions of the surface. These results indicate that a redistribution of the phase composition (migration of excess Ti + N interstitially located nitrogen to the surface) occurs in the oversaturated lattice with increasing temperature. Based on the observations and results presented here, we conclude that the more saturated the lattice is (higher applied fluence), the more radiation defects are generated, which lead to the redistribution of Ti + N after radiation defects. This is also indicated by the fact that the concentration of Ti + N has an increasing tendency in the shallower surface region and, conversely, a decreasing tendency in the deeper region, which corresponds to the vacancy concentration gradient from the TRIM simulation presented in our previous work [18]. The effect of point defects generated during ion implantation on nitrogen redistribution has also been observed in nitrogen-implanted, Ti-modified austenitic steel [22]. The distribution of radiation defects (especially vacancies) in the lattice after implantation was considered by the authors to be the origin of the observed redistribution of nitrogen to the surface [18,22]. Undoubtedly, annealing time and temperature can have a significant effect on structural changes. However, comparable quantitative changes in phase composition are observed even though the annealing time increased from 3 h for fluence 3∙10^17^ cm^−2^ to 6 h for fluence 6∙10^17^ cm^−2^ and to 11 h for fluence 9∙10^17^ cm^−2^. A higher temperature undoubtedly leads to an acceleration of redistribution and to a greater representation of Ti + N in the region closer to the surface.

**Figure 4:**
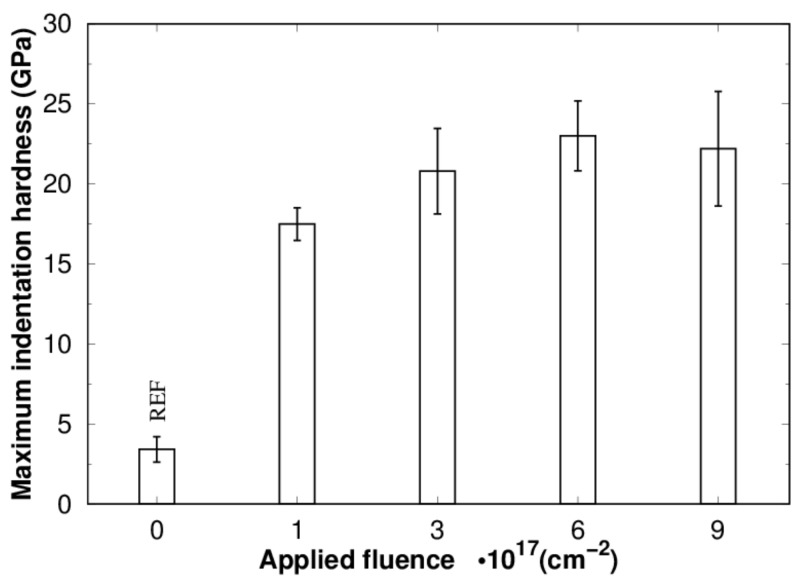
Maximum surface hardness of titanium implanted with nitrogen ions with fluences in the range of 1–9 10^17^ cm^−2^ without post-implantation annealing evaluated from hardness depth profiles from a depth of up to 25 nm.

Figure 4 shows the evolution of the maximum surface hardness of titanium implanted with nitrogen ions with fluences in the range from 1·10^17^ to 9·10^17^ cm^−2^. The results indicate that the surface hardness increases with the fluence of the implanted ions and follows the increasing trend of the maximum nitrogen concentration (Figure 1). The changing nitrogen concentration gives rise to the structural phases typical for the Ti-N system according to the phase diagram [12]. Quantitative phase analysis indicates that the origin of the surface hardening is a mixture of Ti(+N) and TiN. No Ti_2_N phase was found in the structure of the implantation zone even after post-implantation annealing. Many studies have shown that TiN phase formation is preferred over Ti_2_N phase formation in low temperature nitrogen ion implanted titanium [7,16]. Remarkable surface hardening of approx. 17 GPa was found for titanium implanted with a fluence of 1·10^17^ cm^−2^, where Ti(+N) is the major structural phase of the implantation zone (Table 1). An increase in surface hardness to values of approx. 21, 23 and 22 GPa was found for fluences of 3, 6 and 9·10^17^ cm^−2^, respectively. The observed increase in surface hardness is related to increased formation of TiN, which becomes the main phase of the implantation zone due to the high concentration of nitrogen, when such high fluences are applied. The surface hardness values obtained on titanium implanted with fluences of 3·10^17^ cm^−2^ and higher show higher deviation, probably caused by saturation defects, leading to the structural inhomogeneities and increased data variance during the measurements. The significant representation of defects, cracks and pores in the titanium surface implanted with the highest fluence of 9·10^17^ cm^−2^ probably leads to nitrogen effusion, significant inhomogeneities in the integrity of the structure and a decrease in hardness. A similar trend of surface hardening after high fluence N ion implantation was observed by Zhang et al. on martensitic stainless steel [23]. They observed decreasing surface hardness above a fluence of 5·10^17^ cm^−2^.

**Figure 5:**
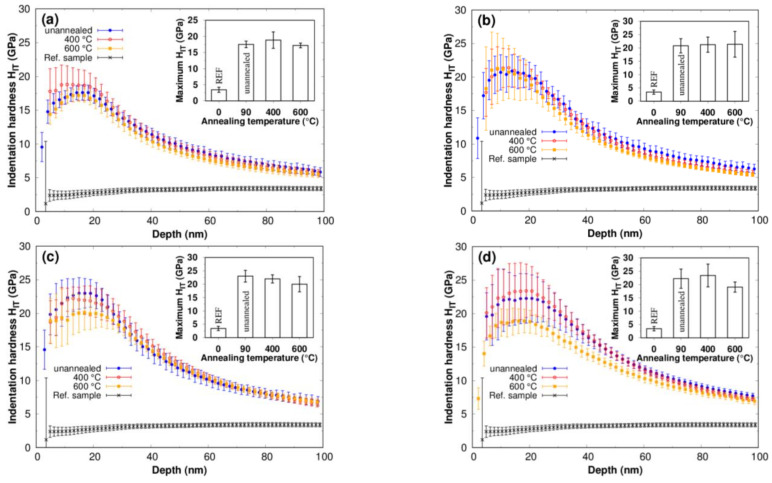
Depth profiles of indentation hardness measured on titanium implanted with nitrogen ions with fluences of (**a**) 1∙10^17^ cm^−2^, (**b**) 3∙10^17^ cm^−2^, (**c**) 6∙10^17^ cm^−2^ and (**d**) 9∙10^17^ cm^−2^ and post-implantation annealed at temperatures of 400 °C and 600 °C. The inserts show the maximum indentation hardness evaluated from hardness depth profiles from a depth of up to 25 nm.

The effect of post-implantation annealing on the surface hardness of nitrogen ion-implanted titanium is shown in Figure 5. The surface hardness seems to be stable after annealing for the titanium implanted with fluences of 1 and 3·10^17^ cm^−2^ (Figure 5a,b). The discrepancies in surface hardness between the annealed state (400 °C and 600 °C) and the unannealed state (90 °C—the maximum temperature during ion implantation) are insignificant within the data variance. The annealing-induced changes in surface hardness are apparent in titanium implanted with higher fluences of 6 and 9·10^17^ cm^−2^ (Figure 5c,d). There is a reduction in surface hardness with the temperature of annealing. A significant decrease in surface hardness was found after annealing at 600 °C. The rearrangement of nitrogen in the interstitial positions of Ti(+N) and its decreasing concentration in the deeper region without the possibility of increasing TiN in the oversaturated implanted zone thus apparently leads to a decrease in hardness at fluences higher than 3, where significant oversaturation occurs. The amount of Ti(+N) in the structure is proportional to the internal stress [10,17,24]. This hardness decrease mechanism was observed on the annealed titanium implanted with an extreme fluence higher than 1·10^18^ cm^−2^ [10,17]. Redistribution of Ti(+N) to the surface is also observed at lower fluences of 1 and 3·10^17^ cm^−2^. However, here, the decrease in hardness due to the rearrangement of nitrogen in interstitial positions is apparently compensated by the additional formation of TiN, as indicated by XRD phase analysis. The results presented here demonstrate that the presence of Ti(+N) enhances the hardening and plays a significant role during post-implantation annealing on the reduction in surface hardness. However, the surface preserves a high level of hardening after post-implantation annealing. This, together with the phase analysis, confirms that the TiN phase is stable during annealing up to 600 °C. The stability of TiN at 600 °C was also observed by Berberich et al. [16].

## 4. Conclusions

Titanium samples were implanted with 90 keV nitrogen ions with fluences of 1, 3, 6 and 9∙10^17^ cm^−2^. Post-implantation annealing at temperatures of 400 °C and 600 °C was performed in order to study its effect on hardness stability. We have demonstrated that fluence of 3∙10^17^ cm^−2^ and higher led to oversaturation of the implantation zone and to saturation defects on the surface. A remarkable increase in surface hardness was found after ion implantation due to the formation of a mixture of TiN and Ti(+N). An oversaturated surface, saturation defects and the phase composition of the implantation zone, together with the applied fluence, have an impact on the hardness stability after post-implantation annealing. Hardness stability after post-implantation annealing was observed in titanium implanted with nitrogen with fluences that do not lead to significant oversaturation (fluence of 1 and 3∙10^17^ cm^−2^). Conversely, a decrease in indentation hardness was found after the annealing of samples implanted with fluences of 6 and 9∙10^17^ cm^−2^, for which there is significant oversaturation. Annealing-induced redistribution of interstitial nitrogen Ti(+N) in the implantation zone and the ability to form additional TiN seems to be the origin of the observed behavior. While Ti + N redistribution is accompanied by the formation of TiN at lower fluences, no additional formation of TiN in the oversaturated implantation zone was observed at high fluences. The results presented here show that post-implantation annealing/heating with temperatures up to 600 °C can lead to a specific change in surface hardness and can thus affect the functional performance of the titanium components. It has been shown that the fluences of the implanted ions and the level of oversaturation play a significant role in annealing-induced hardness degradation.

## Figures and Tables

**Table 1:** Phase composition (wt.%) of a titanium surface implanted with various fluences of nitrogen ions and post-implantation annealed at 400 °C and 600 °C. XRD measurement with incident angles 0.5° and 2° was used to investigate the changes in the phase composition of the shallow and deeper region of the modified surface.

	Fluence 10^17^ (cm^−2^)	1			3			6			9		
	Annealing Temperature (°C)	-	400	600	-	400	600	-	400	600	-	400	600
Incident angle 0.5°	TiN	15	24	18	85	87	82	85	84	82	82	80	84
	Ti(+N)	81	69	80	12	13	17	11	16	17	12	19	16
	α-Ti	4	7	2	3	0	1	4	0	1	6	1	0
Incident angle 2°	TiN	1	7	3	50	51	49	49	51	49	52	48	53
	Ti(+N)	65	55	61	38	37	35	37	36	34	34	31	27
	α-Ti	35	37	36	12	12	16	14	13	17	14	21	20

## Data Availability

Data are available from the corresponding author upon reasonable request.
